# Effect of Graphene Family Materials on Multiple Myeloma and Non-Hodgkin’s Lymphoma Cell Lines

**DOI:** 10.3390/ma13153420

**Published:** 2020-08-03

**Authors:** Barbara Strojny, Sławomir Jaworski, Irena Misiewicz-Krzemińska, Isabel Isidro, Elizabeta A. Rojas, Norma C. Gutiérrez, Marta Grodzik, Piotr Koczoń, André Chwalibog, Ewa Sawosz

**Affiliations:** 1 Department of Nanobiotechnology and Experimental Ecology, Institute of Biology, Warsaw University of Life Sciences, 02-786 Warsaw, Poland; slawomir_jaworski@sggw.edu.pl (S.J.); marta_grodzik@sggw.edu.pl (M.G.); ewa_sawosz_chwalibog@sggw.edu.pl (E.S.); 2 Hematology Department, Institute of Biomedical Research of Salamanca (IBSAL), University Hospital of Salamanca, 37007 Salamanca, Spain; irenamk@usal.es (I.M.-K.); imisidroh@gmail.com (I.I.); elirr@usal.es (E.A.R.); normagu@usal.es (N.C.G.); 3 Cancer Research Center-IBMCC (USAL-CSIC), 37007 Salamanca, Spain; 4 Department of Chemistry, Institute of Food Science, Warsaw University of Life Sciences, 02-776 Warsaw, Poland; piotr_koczon@sggw.edu.pl; 5 Department of Veterinary and Animal Sciences, University of Copenhagen, Groennegaardsvej 3, 1870 Frederiksberg, Denmark; ach@sund.ku.dk

**Keywords:** graphene, graphene oxide, nanostructures, cancer cells, multiple myeloma, lymphoma, non-adherent cells

## Abstract

The interest around the graphene family of materials is constantly growing due to their potential application in biomedical fields. The effect of graphene and its derivatives on cells varies amongst studies depending on the cell and tissue type. Since the toxicity against non-adherent cell lines has barely been studied, we investigated the effect of graphene and two different graphene oxides against four multiple myeloma cell lines, namely KMS-12-BM, H929, U226, and MM.1S, as well as two non-Hodgkin lymphoma cells lines, namely KARPAS299 and DOHH-2. We performed two types of viability assays, MTT (3-(4,5-dimethylthiazol-2-yl)-2,5-diphenyltetrazolium bromide conversion) and ATP (adenosine triphosphate detection), flow cytometry analysis of apoptosis induction and cell cycle, cell morphology, and direct interaction analysis using two approaches—visualization of living cells by two different systems, and visualization of fixed and dyed cells. Our results revealed that graphene and graphene oxides exhibit low to moderate cytotoxicity against cells, despite visible interaction between the cells and graphene oxide. This creates possibilities for the application of the selected graphene materials for drug delivery systems or theragnostics in hematological malignancies; however, further detailed studies are necessary to explain the nature of interactions between the cells and the materials.

## 1. Introduction

Since the discovery of graphene, the interest around it has been growing, leading to the production and synthesis of similar carbon materials, including those derived from natural graphene, also known as pristine [1]. Among them, the most broadly studied are different forms of oxidized graphene, namely graphene oxides, obtained by varying chemical synthesis methods, as well as their reduced forms, namely reduced graphene oxides. Other examples include graphite, which is the blank material for graphene and graphite nanoparticles, as well as carbon nanotubes, which have a structure that is basically rolled-up graphene [2]. All of these graphene-related materials have a characteristic honeycomb network of carbon atoms with delocalized π electrons and varied amounts of oxygen-containing groups, leading to a large active surface and easy functionalization. They have potential in various areas, such as nanoelectronics [3], composites, photovoltaics [4,5], and the biomedical field, including tissue engineering, biosensors [6,7], antibacterial [8,9] and antiviral [10] agents, functional dressings [11,12], cancer diagnostic tools [13], treatments [14], and drug delivery systems [15].

Determining potential biomedical applications still requires a lot of studies since the effect of the graphene family materials on cells, tissues, and whole organisms is ambiguous and depends on many factors, such as the type of material, its size, the route of exposure or administration, and the cell or tissue type [16]. So far, it has been demonstrated that graphene has anticancer properties [17,18]; however, its ability to induce apoptosis or cell cycle arrest, as well as its general toxicity, depend on the flake size and type of tissue, inducing different responses to the contact with graphene. It has been proposed that use of graphene’s ability to interrupt cell membranes can be employed in super-sensitive sensors for cancer cell detection in blood samples [19]. Due to its oxygen content and more hydrophilic nature, graphene oxide has been mostly described as a biocompatible material with low toxicity [20]. However, these properties may also depend on the above-mentioned factors, and the graphene oxide might be cytotoxic against cells in in vitro conditions [21,22]. It has also been demonstrated that graphene oxide can strongly bind to biomolecules, leading to changes in their molecular activity, for example inhibiting enzyme reaction, similarly to carbon nanotubes [23,24].

The studies of cancer performed so far concern solid tumors and adherent cell cultures [25], while there is little known about the interaction with non-adherent cells, which are commonly derived from hematological cancers. Such investigation is needed since graphene materials might interact differently with adherent cells and non-adherent cells in suspension, which is important when designing intelligent graphene-based nanoplatforms for cancer treatment, detection, and imaging. In this study, we used six cell lines derived from distinct types of hematological malignancies, namely multiple myeloma (MM) and non-Hodgkin lymphoma (NHL).

Multiple myeloma is a bone marrow malignancy derived from terminally matured B cells and plasma cells, that due to the abnormal accumulation that leads to hematopoietic function failure, anemia, and increased occurrence of fractures [26]. Despite advances in studies on the outcomes of MM, it remains an almost incurable disease, with the recurrence of the disease being the most critical issue [27,28]. Lymphomas are a heterogenous group of malignancies, with a higher incidence of NHL, and both are derived from B and T lymphocytes [29]. Thus, MM and NHL are distinct types of hematological cancer. Here, the effect of natural graphene and two types of graphene oxide on four MM and two NHL cell lines was investigated, revealing low to moderate toxicity and lack of apoptosis induction with possible binding to cells at the same time. The obtained results suggest potential applications of selected graphene materials as drug carriers for hematological malignancies or theragnostics.

## 2. Materials and Methods

### 2.1. Graphene Family Materials

Natural graphene (GN) flakes in powder form were obtained from SkySpring (Houston, TX, USA), where they were produced by an exfoliation method from 100% graphite. The GN powder contained >99.5% carbon. According to the manufacturer, the specific surface area of the flakes was 120–150 m^2^/g, with an average particle diameter of 15 μm and thickness of 6–8 nm. Graphene oxide (GO) platelets were obtained from Nanopoz (Poznan, Poland). They were produced by a modified Hummers’ method, described earlier [30]. Apart from carbon, the GO flakes contained 35–49% oxygen, 1–4% hydrogen, <2% sulfur, and 1% nitrogen. GO was delivered in the form of a hydrocolloid at a concentration of 4000 mg/L. According to the manufacturer, the GO platelet size was 5–30 µm and the thickness of a single layer was approximately 1 nm. Nano-sized graphene oxide (nGO) was obtained in the form of a powder from the Institute of Electronic Materials Technology (Warsaw, Poland), where it was produced by a modified Hummers’ method [31] from graphite nanoplatelets (Asbury Carbons, Asbury, NJ, USA). The average size of the nGO nanoplatelets was 8–25 nm. Analysis of Raman spectra for nGO was previously provided [32].

GN and nGO were dispersed in ultrapure water (1000 mg/L). GO was diluted to a concentration of 1000 mg/L and sonicated at 550 W/m^2^ for 1 h in an ultrasonic bath (Ultron, Olsztyn, Poland). From the 1000 mg/L stock solutions, the following 10× concentrated solutions were prepared in ultrapure water: 50, 100, 200, and 500 mg/L.

### 2.2. Physicochemical Analysis of Natural Graphene, Graphene Oxide, and Nano-Sized Graphene Oxide

The size and shape of the GN, GO, and nGO (at a concentration 50 mg/L in ultra-pure water) were inspected using a JEM-1220 transmission electron microscope (JEOL, Tokyo, Japan) at 80 KeV, with a Morada 11 megapixels camera (Olympus Soft Imaging Solutions, Münster, Germany).

Zeta potential measurements were performed by the microelectrophoretic method with Smoluchowski approximation, and size distribution with hydrodynamic diameter was measured by the dynamic light scattering technique using a Zetasizer Nano-ZS90 analyzer (Malvern, Worcestershire, UK). Each measurement was performed after 120 s of stabilization at 25 °C and in triplicates.

Fourier transform infrared spectroscopy (FT-IR) spectra were registered in the middle infrared range of 4000–400 cm^−1^ with use of Perkin Elmer System 2000 spectrometer (PerkinElmer Inc., Waltham, MA, USA). Initially, the spectrum of background containing bands generated by vibrations and rotations of CO_2_ and H_2_O_(g)_ always present in air was registered. Next, a drop of liquid sample (nGO, GO) was placed on round KRS plate (5 cm diameter) and pressed with another the same size plate to form a film. Then transition spectrum (ratio of working sample to background signals) of liquid sample was registered with 20 scans and 4 cm^−1^ resolution. In the case of solid-state samples (GN) powder was mixed with KBr crystals for 5 min in the laboratory mill in the ratio of 1:300 (m/m) to obtain fine powder. Powder obtained was placed in the pellet maker and pressed with a 5-ton hydraulic laboratory press for 1 min to obtain 13 mm diameter, as thin as possible pellet. Pellet was placed into a dedicated holder which was placed in the measuring chamber of spectrometer. Then sample was radiated with infrared radiation from 4000–400 cm^−1^ spectral range to collect data for spectrum.

Microscopic images from scanning electron microscope (SEM) were taken using scanning electron microscopy with cold field emission (FE-SEM) model Hitachi SU8010 (Hitachi Ltd., Tokyo, Japan). Powdered natural graphene (GN) was placed directly onto conductive carbon tape. Excess powder was removed using a photographic pear. nGO and GO in the aqueous suspension were transferred with a pipette to a conductive carbon tape. Microscopic observations were carried out after evaporation of the water. None of the samples were coated with a conductive layer. The images were taken in the SE (secondary electrons) mode at an accelerating voltage of 10 kV. To determine the purity of GN, nGO, and GO, elemental point analysis (EDS) was performed using a NORAN system seven X-ray energy dispersion spectrometer and SSD detector (Thermo Scientific, Waltham, MA, USA).

### 2.3. Cell Lines

Six cell lines were used in the study: four multiple myeloma (KMS-12-BM, H929, U266, and MM.1S) and two NHL T and B lymphocyte-derived (KARPAS299 and DOHH-2, respectively) cell lines. KMS-12-BM, U266, KARPAS299, and DOHH-2 were obtained from Leibniz Institute DSMZ (German Collection of Microorganisms and Cell Cultures GmbH, Leibniz, Germany), and H929 and MM.1S were obtained from ATCC (American Type Culture Collection, Manassas, VA, USA). Cells were cultured in RPMI 1640 medium (Gibco; Thermo Fisher Scientific, Waltham, MA, USA), supplemented with 20% fetal bovine serum (FBS; Gibco) and 1% antibiotic mix (Gibco) of penicillin (100 U/mL) and streptomycin (100 mg/mL), and they were maintained at 37 °C in a humidified atmosphere containing 5% CO_2_. All cells are characterized by the type of suspension culture, except for MM.1S, which is a mixed semi-adherent cell line (suspension with lightly attached cells).

For all assays, cells were seeded at a density of 5 × 10^5^ cells/mL. For the viability tests, they were seeded on a 96-well microplate (Corning) in 90 μL of medium per well. For flow cytometry and morphology analyses, cells were seeded on a 12-well plate in 900 μL of medium per well. Two hours after seeding 10× concentrated solutions of GN, GO and nGO were introduced into the wells (10 μL/well on a 96-well plate or 100 μL/well on a 12-well plate), obtaining final nanomaterial concentrations of 5, 10, 20, 50, and 100 mg/L directly in the culture media with cells. Cells were incubated for 24 h before the tests.

### 2.4. Viability Assays

For evaluation of cell viability after treatment with GN, GO, and nGO, two kinds of tests were employed: MTT and ATP assays. All the tests were performed in three independent experiments.

The MTT colorimetric assay is based on a conversion of yellow, soluble tetrazolium salt to purple formazan crystals by mitochondrial succinate dehydrogenase of active cells. MTT (Sigma Aldrich, St Louis, MO, USA) was dissolved in PBS at a concentration of 5 mg/mL and 15 μL was added per each well. After 3 h of incubation at 37 °C, the crystals were dissolved in the solubilization detergent (isopropanol, 0.01 N HCl). Before readings, plates were centrifuged (5 min, 400× *g*) in order to remove clusters of nanostructures and the supernatant was transferred to a new plate. Spectrophotometer readings were performed at a wavelength of 570 nm in a microplate reader (Tecan Group Ltd., Männedorf, Switzerland).

The ATP luminometric CellTiter-Glo assay (Promega, Madison, WI, USA) provides a determination of the number of viable cells by quantitation of ATP in active cells, which converts a substrate into a luminescent product. For this assay, cells were seeded on opaque-walled white multiwell plates (Promega). After equilibration to room temperature, CellTiter-Glo Buffer was mixed with the substrate and then 100 μL of the solution was added to each well. After 2 min of shaking, plates were incubated for 10 min in the dark to stabilize the signal. Then, the luminescence was measured with the microplate reader (BioTek, Winooski, VT, USA).

For both assays, the results are presented as the % of control and were calculated from the formula

$$\%\;\mathrm{of}\;\mathrm{control}=\frac{A}{C}\times 100\%$$

where A is the absorbance or luminescence in the treated group and C is the mean absorbance or luminescence in the control group.

### 2.5. Apoptosis Assay

Apoptosis induction was evaluated using the Annexin V FITC Apoptosis Detection Kit (Immunostep, Salamanca, Spain) and analyzed by flow cytometry. The test relies on double fluorescent staining, where annexin V conjugated with FITC (AnnV) binds to the phosphatidylserine that is externally exposed during apoptosis, and propidium iodide (PI) binds to the nucleic acids in cells with damaged membranes, allowing for bivariate analysis. The tests were performed in duplicate and repeated in three independent experiments.

After incubation with GN, GO, and nGO (20 mg/L) cells were harvested and transferred to cytometric tubes, washed twice with Annexin V Binding Buffer (10 mM HEPES/NaOH, pH 7.4; 140 mM NaCl; 2.5 mM CaCl_2_), and then resuspended in 100 μL of the same buffer. Five microliters of AnnV and 2.5 μL of PI were added to each tube, and the cells were incubated at room temperature for 15 min in the dark. Then, 400 μL of the buffer was added to each tube, and the cells were analyzed with a BD FACSCalibur™ cytometer (Becton Dickinson, Franklin Lakes, NJ, USA), recording 20,000 events per sample. Dot plots were generated and analyzed using Flowing Software 2.5.1 (University of Turku, Turku, Finland).

### 2.6. Cell Cycle Assay

The cell cycle was analyzed using PI/RNase Solution (Immunostep) dedicated for flow cytometry. This assay is based on the quantification of the DNA content in cells and the distribution of a cell population among the three phases of the cell cycle (G0/G1, S, and G2/M). PI stains all nucleic acids in fixed cells; thus, RNase is used to eliminate the RNA for the analysis. The tests were performed in duplicates and repeated two independent times.

After incubation with GN, GO, and nGO (20 mg/L) cells were harvested and transferred to cytometric tubes and centrifuged to remove the medium. Then, cells were fixed with 70% ethanol at 4 °C for 30 min (200 μL per tube) and washed in PBS. The cell pellet was resuspended in the residual liquid with an additional 0.5 mL of the PI/RNase solution and incubated for 15 min at room temperature in the dark before the analysis on a BD FACSCalibur™ cytometer (Beckton Dickinson). Two hundred thousand events were counted, and plots were generated and analyzed using Flowing Software 2.5.1.

### 2.7. Morphology Evaluation

For cell morphology evaluation and to visualize the interaction between cells and carbon nanostructures, two approaches were used: imaging of viable cells with no staining and imaging of fixed stained cells. For live imaging, two systems were used, namely the EVOS Cell Imaging Station (Life Technologies, Carlsbad, CA, USA) and the Nikon Eclipse TE2000-E microscope in phase contrast (Nikon, Tokyo, Japan).

For imagining after staining, cells were transferred to the tubes attached to the microscopic slides and centrifuged in order to deposit cells on the slides using a Cytospin 4 cytocentrifuge (Thermo Scientific, Waltham, MA, USA). Cells were covered with May-Grünwald stain, and after 3 min the stain was diluted with an equal amount of PBS. After 3 min, the stain was replaced with Giemsa stain (diluted 1:20 in distilled water). After 20 min, the stain was removed and the slides were washed thoroughly with distilled water. Images were recorded using an Olympus BX51 microscope equipped with a DP70 camera (Olympus, Tokyo, Japan).

### 2.8. Statistical Analysis

Cell viability results were analyzed by mono-factorial analysis of variance with the post-hoc Dunnett test. Results from apoptosis assays were analyzed by the unpaired *t*-test. For *p*-values that were <0.05 were considered significant. The analyses were performed using Statgraphics Centurion version XVI software (Warrenton, VA, USA).

## 3. Results

### 3.1. Physicochemical Analysis of GN, GO, and nGO

**Figure 1:**
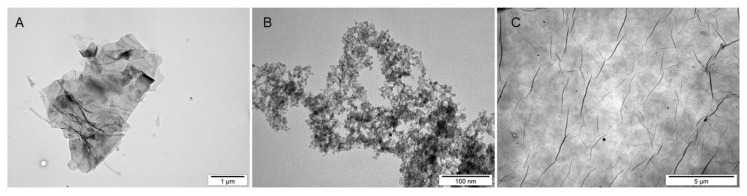
TEM images of the graphene family materials used in the experiments. (**A**) GN (1 µm scale bar), (**B**) nGO (100 nm scale bar), and (**C**) GO (5 µm scale bar).

Transmission electron microscopy (TEM) analysis was performed to evaluate the morphology of the nanostructures. The analysis confirmed the data provided by the manufacturers, revealing the existence of flakes between 1 and 5 µm in GN; however, smaller, irregular flakes were also present (Figure 1A), while the size of the particles for nGO was below 25 nm, with a rather regular shape (Figure 1B). For GO, wide platelets were observed, forming a homogenous layer with visible folds (Figure 1C). Different scales were applied to the pictures in order to present the morphology in the most optimal way in terms of the size, general shape, folding, and edge exposition.

**Figure 2:**
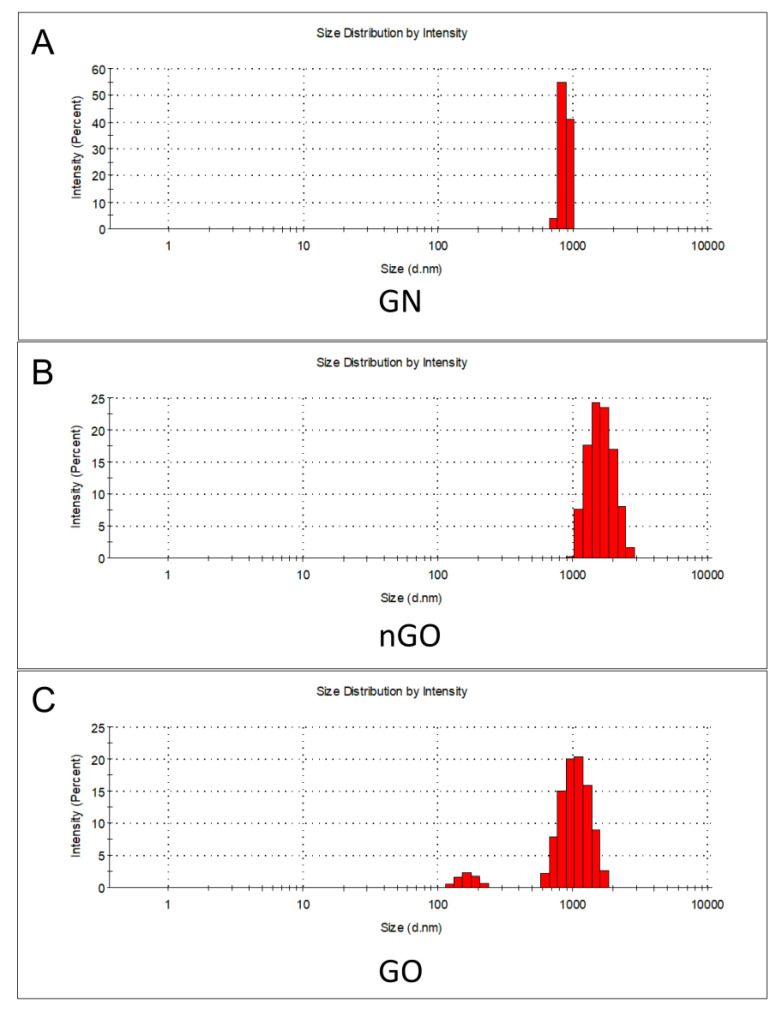
Histograms of the size distribution for selected measurements of the hydrodynamic diameter. Intensity (%) in relation to size (hydrodynamic diameter, nm; logarithmic scale). (**A**) GN, (**B**) nGO, and (**C**) GO.

The zeta potential for both graphene oxides was negative: −13.7 mV for nGO, indicating moderate to low stability of the hydrocolloid, and −64.3 mV for GO (Table 1), indicating high stability of the hydrocolloid, which was in accordance with the observation of the samples during storage in the laboratory. For GN, the zeta potential was 11.0 mV, confirming the moderate to low stability. The average hydrodynamic diameter was high (>1 µm for nGO and GO; >5 µm for GN), confirming the presence of larger flakes or their aggregates, especially for nGO, because it was clear from the TEM analysis that the flakes were below 25 nm. However, it should be noted that measurements of the average hydrodynamic diameter are most suitable for globular nanostructures. Therefore, they are not accurate for graphenoid flakes, giving high deviations but a general insight into their nature in hydrophilic medium (Table 1, Figure 2).

**Figure 3:**
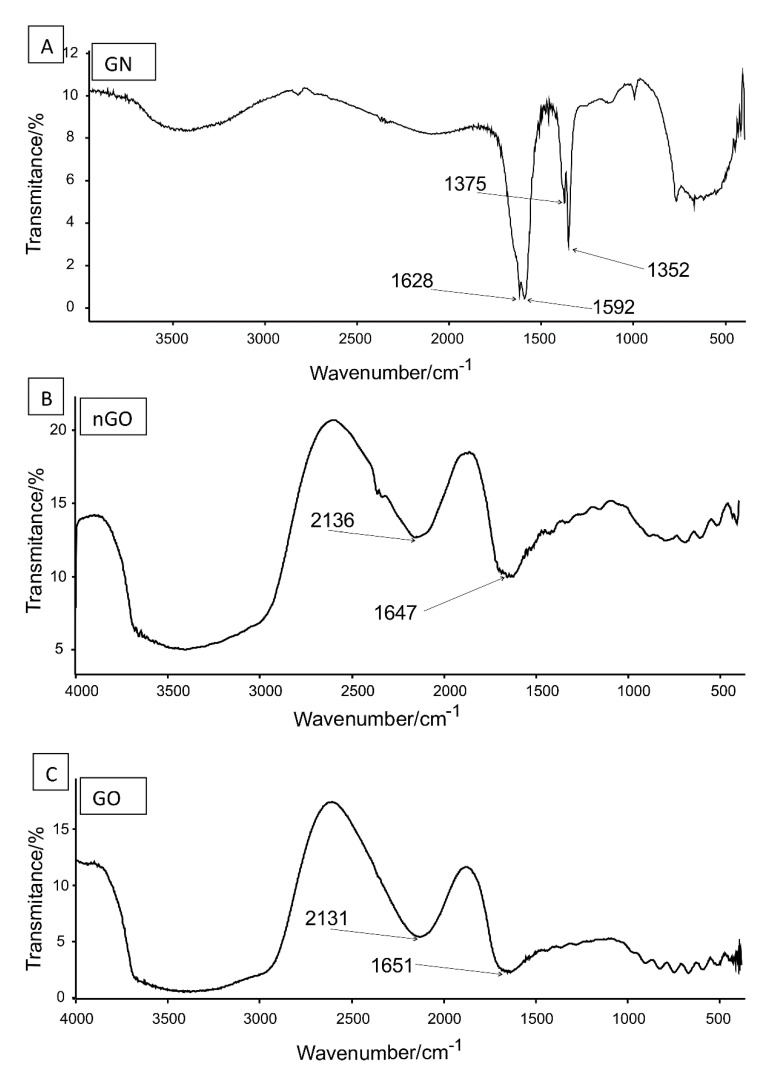
FT-IR spectra of GN (**A**), nGO (**B**), and GO (**C**) with characteristic bands marked. Detailed explanation in the main text.

In FT-IR spectrum of GN (Figure 3A) there were four characteristic bands, two located in spectral region ~1600 cm^−1^, and two in region ~1360 cm^−1^. Bands at 1628 and 1592 are assigned to stretching of C=C bond (double carbon–carbon bonds with sp^2^ hybridized carbon atoms). The energy of such bonds is around 600 kJ × mol^−1^. Both bands in lower spectral region, i.e., 1375 and 1352 are assigned to stretching of C–C bonds (single carbon–carbon bonds with sp^3^ hybridized carbon atoms). Energy of this type of bonds is around 350 kJ × mol^−1^. Bands around 770–400 cm^−1^ are not assigned to any specific vibrations. The wide band of low intensity located around 3500 cm^−1^ is most probably generated by O–H stretches. FT-IR spectra for GO and nGO were highly similar (Figure 3B,C). Two characteristic bands were located at 2131 and 1651 cm^−1^ respectively. Bands at higher location are assigned to C=C (triple bonds between carbon atoms that both are of sp hybridization) stretches. This band is very characteristic and it is located around 2200–2100 cm^−1^. Energy of triple bond is around 850 kJ × mol^−1^. The band located at 1651 cm^−1^ clearly presented in Figure 3 is generated by stretches of C=C bonds. Wide band at around 3500 cm^−1^ is most probably generated by stretches of O–H bonds. A shoulder around 3000 cm^−1^ is again most probably due to C–H stretches. Those stretches are characteristic for C–H when carbon is of sp^2^ hybridization (connected to another carbon by double bond). If the C–H bond is of the sp hybridized carbon (involved in triple bond) bands are located rather more left around 3300 cm^−1^ that is not clearly seen in spectrum registered as covered by intense and wide O–H stretches generated band.

**Figure 4:**
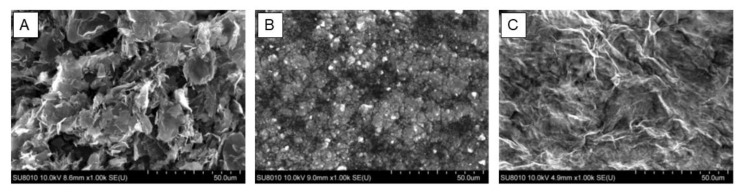
SEM images of the graphene family materials used in the experiments. (**A**) GN, (**B**) nGO, and (**C**) GO.

**Figure 5:**
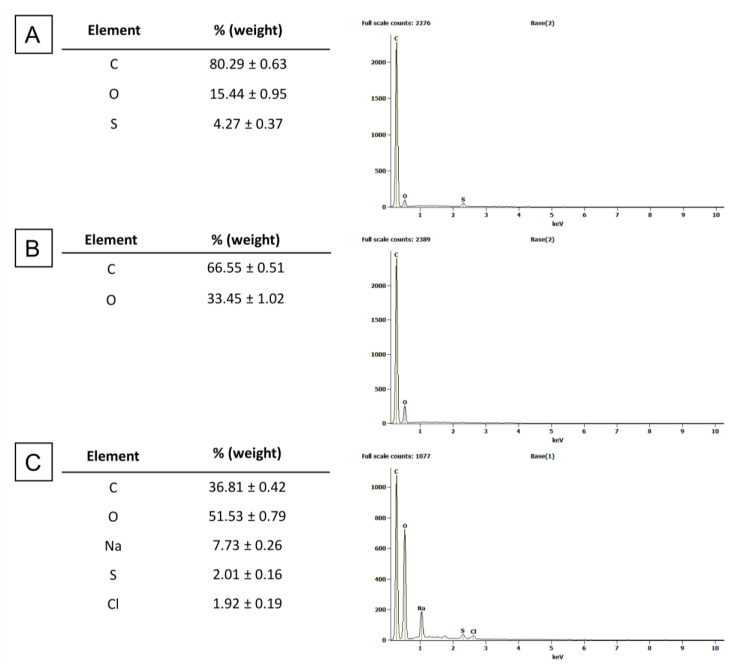
Elemental composition of the carbon materials used in the experiments -GN (**A**), nGO (**B**), and GO (**C**) determined by SEM/EDS method.

Images taken under SEM present additionally the general morphology of the studies materials (Figure 4). Images show characteristic sharp edges of GN flakes, the formation of large GO surface with thin layers and the formation of clusters formed by nanoflakes of nGO after water evaporation. Elemental analysis showed the dominant presence of carbon and oxygen in GN, nGO, and GO. In GN and GO sulphur was present, additionally in GO sodium and chlorine were detected (Figure 5). Hydrogen was not possible to detect using this method.

### 3.2. Viability Test

**Figure 6:**
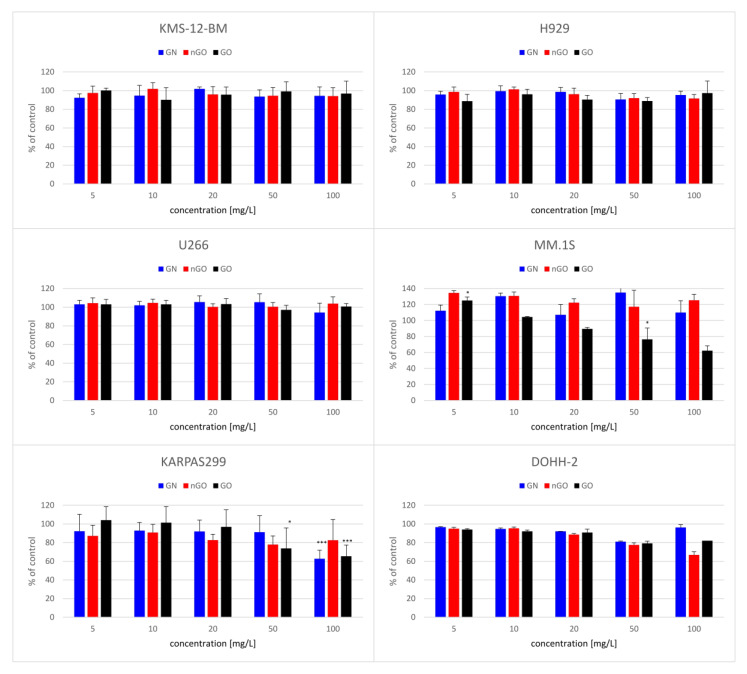
Viability of the cells after 24 h of exposure to the graphene family materials, determined by MTT assay. Results are presented as the % of control (mean with standard variation). A statistically significant difference between a group and the control was marked as * *p* < 0.05, *** *p* < 0.001. Blue bars, GN; red bars, nGO; black bars, GO.

**Figure 7:**
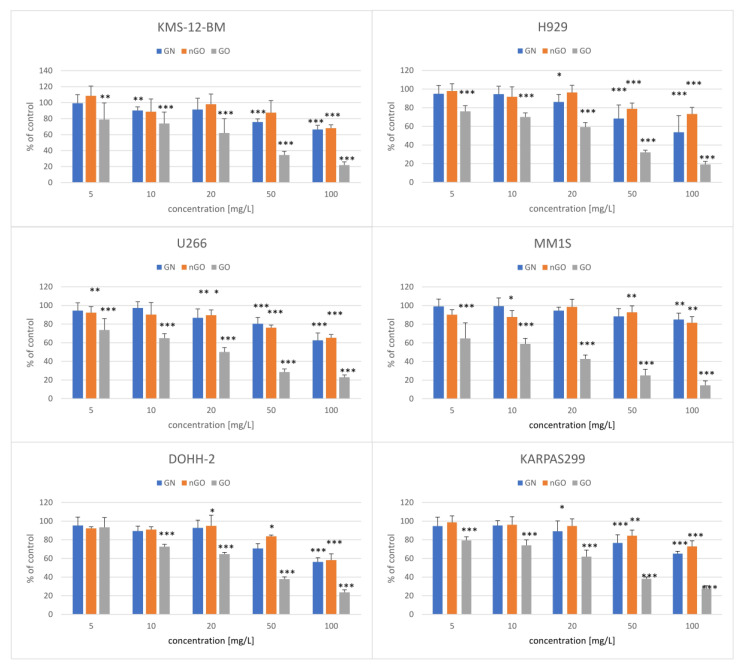
Viability of cells after 24 h of exposure to the graphene family materials, determined by the ATP assay. Results are presented as the % of control (mean with standard variation). A statistically significant difference between a group and the control was marked as * *p* < 0.05, ** *p* < 0.01, *** *p* < 0.001. Blue bars, GN; orange bars, nGO; grey bars, GO.

The performed viability tests revealed variable effects of graphene family materials on multiple myeloma and lymphoma cell lines. Moreover, the results differed between the MTT and ATP tests. Interestingly, the MTT tests showed almost noncytotoxic effect (Figure 6) and even an increase in the MM.1S cell line. On the contrary, the ATP tests revealed a significant decrease in the viability of the cells after incubation with the nanostructures (Figure 7). In general, regardless of the cell line, GO was the most potent in the viability decrease among the used graphene family materials.

### 3.3. Apoptosis Assay

**Figure 8:**
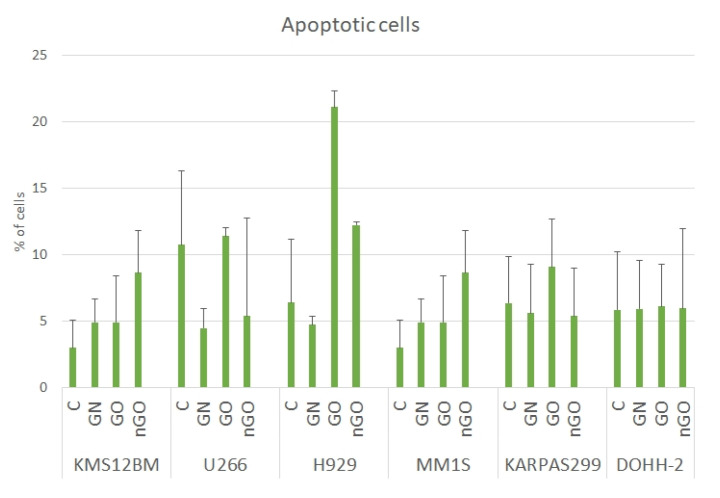
Apoptotic cells in the control groups (C) and in the groups treated with GN, GO, and nGO. Presented as the % of all cells in the group after 24 h of incubation. Results are presented as the mean ± SD. No statistically significant differences were observed.

**Figure 9:**
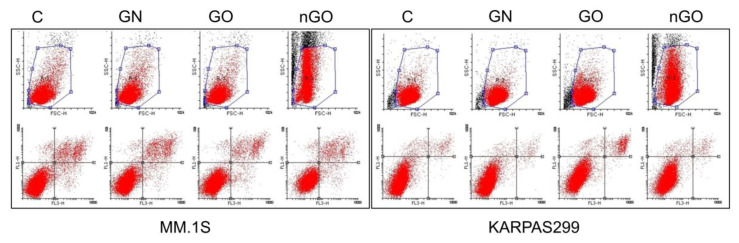
Dot diagrams generated during analysis of the apoptosis assay, where the increase in granularity (SSC values shift) is visible for the nGO-treated groups in selected examples, in comparison to the control group (C), GN, and GO (MM.1S and KARPAS299). Upper row, SSC/FSC plots; lower row, FL1-H (PI)/FL3-H (AnnV) plots.

No significant effect of GN, GO, or nGO on the induction of apoptosis in any of the cell lines was observed. The number of apoptotic cells in the treated and control groups was comparable in all cell lines, varying between 5% and 12%, expect for GO in the H929 cell line, which reached 20% (Figure 8). Interestingly, during the analysis of apoptosis induction, shifts in the granularity of the cells were noticed on the side scatter/forward scatter (SSC/FSC) graphs for the groups treated with nGO. Selected examples are presented in Figure 9.

### 3.4. Cell Cycle Analysis

**Figure 10:**
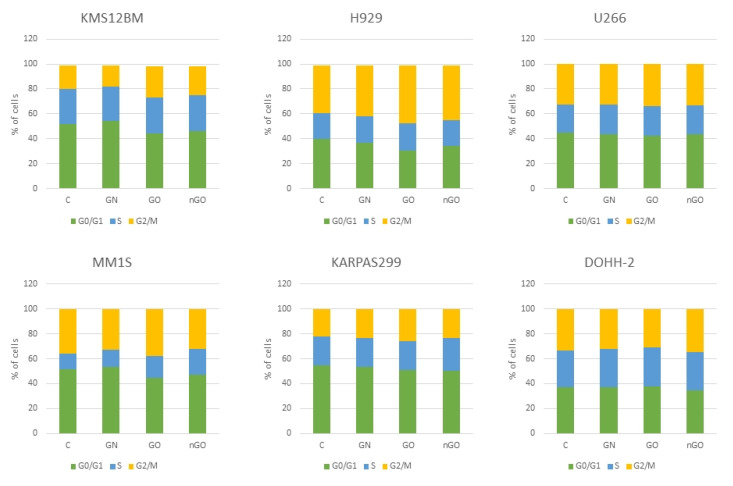
Cell cycle analysis after 24 h of incubation with graphene family materials (GN, GO, and nGO) in comparison to the control groups (C). No significant changes were noted. Green bar, cells in G0/G1 phase; blue bar, cells in S phase; yellow bar, cells in G2/M phase.

The cell cycle analysis did not reveal any effects on the cell cycle after 24 h of incubation with graphenes. The number of cells in GN-, GO-, and nGO-treated groups was comparable to the control groups within each cell line (Figure 10). A slight increase in the cell number in the G2/M phase was noticeable in GO groups in the three myeloma cell lines (KMS-12-BM, H929, and MM.1S).

### 3.5. Morphology Evaluation

**Figure 11:**
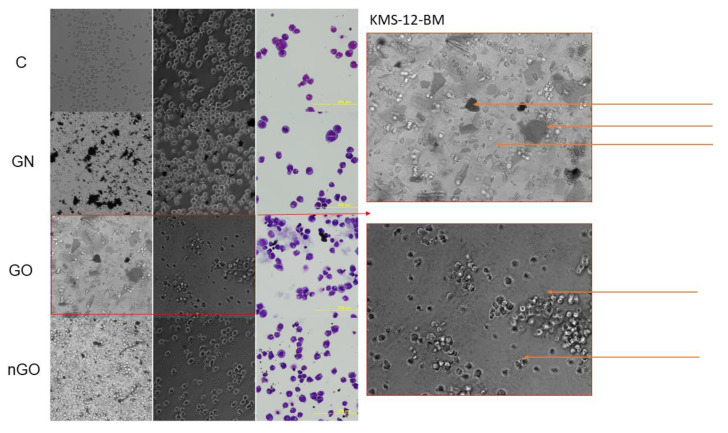
KMS-12-BM cell morphology assessment after 24 h of incubation with graphene family materials (GN, GO, and nGO) in comparison to the control group (C). Live images in bright field and phase contrast and after staining with May-Grünwald and Giemsa (MGG) method. Orange arrows indicate the thick and large GO flakes and the edge of the flake and visible folds in phase contrast. All pictures were taken under the same magnification (200×).

**Figure 12:**
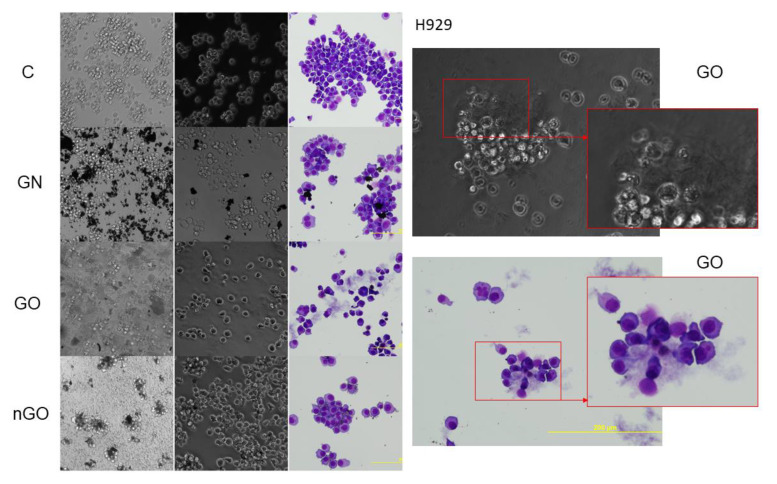
H929 cell morphology assessment after 24 h of incubation with graphene family materials (GN, GO, and nGO) in comparison to the control group (C). Live images in bright field and phase contrast and after staining with May-Grünwald and Giemsa (MGG) method. All pictures were taken under the same magnification (200×). Parts of the selected pictures for the GO group are additionally magnified for clearer visualization of the GO flakes.

**Figure 13:**
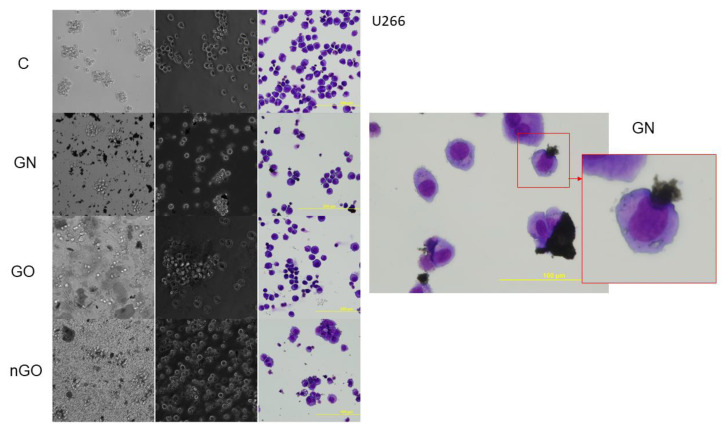
U266 cell morphology assessment after 24 h of incubation with graphene family materials (GN, GO, and nGO) in comparison to the control group (C). Live images in bright field and phase contrast and after staining with May-Grünwald and Giemsa (MGG) method. All pictures in the main panel were taken under the same magnification (200×); the additional picture was taken under 400×. Part of the picture for the GN group is additionally magnified for clearer visualization of the GN flake.

**Figure 14:**
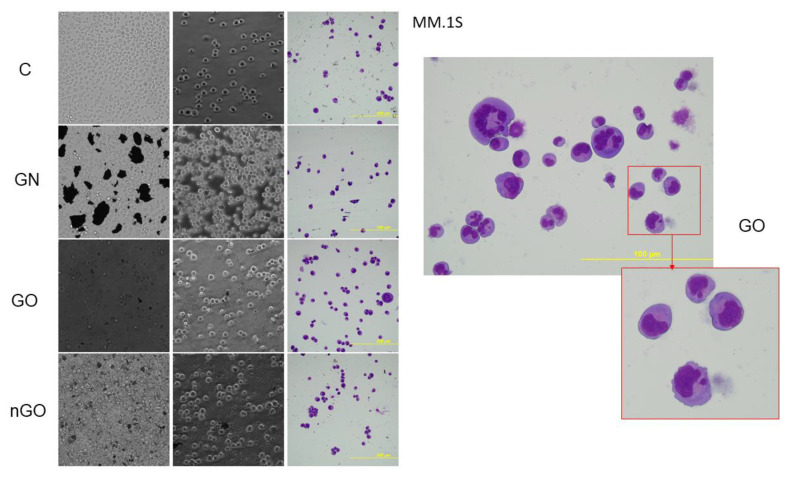
MM.1S cell morphology assessment after 24 h of incubation with graphene family materials (GN, GO, and nGO) in comparison to the control group (C). Live images in bright field and phase contrast and after staining with May-Grünwald and Giemsa (MGG) method. All pictures in the main panel were taken under the same magnification (200×); the additional picture was taken under 400×. Part of the picture for the GO group is additionally magnified for clearer visualization of a pattern, which was characteristic for the GO group, most likely indicating a GO flake.

**Figure 15:**
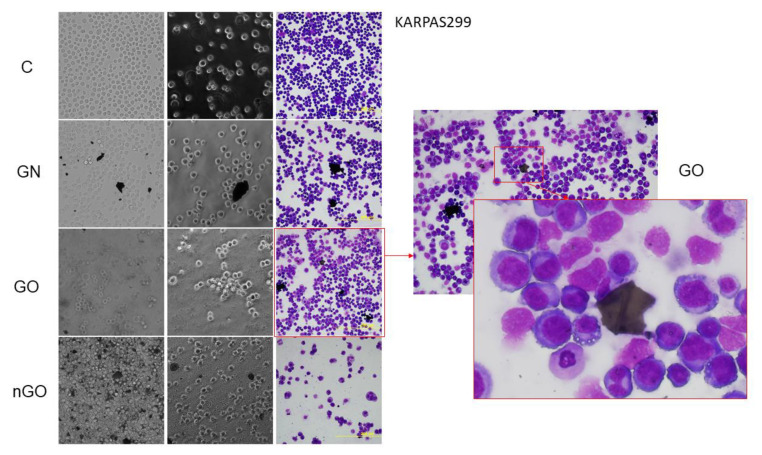
KARPAS299 cell morphology assessment after 24 h of incubation with graphene family materials (GN, GO, and nGO) in comparison to the control group (C). Live images in bright field and phase contrast and after staining with May-Grünwald and Giemsa (MGG) method. All pictures in the main panel were taken under the same magnification (200×). Part of the presented picture for the GO group is additionally magnified for clearer visualization of the large, thick GO flake with a visible fold. These large and thick flakes were rarely visible in the culture.

**Figure 16:**
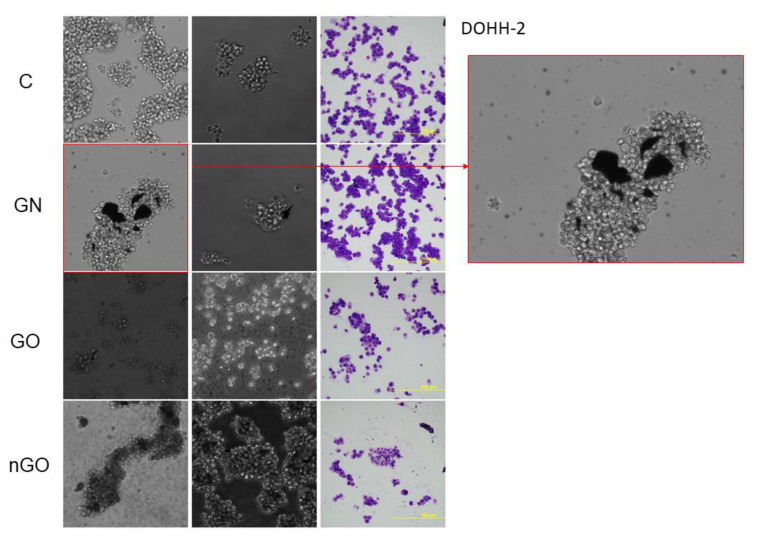
DOHH-2 cell morphology assessment after 24 h of incubation with graphene family materials (GN, GO, and nGO) in comparison to the control group (C). Live images in bright field and phase contrast and after staining with May-Grünwald and Giemsa (MGG) method. All pictures were taken under the same magnification (200×). Part of the presented picture for the GN group is additionally magnified for clearer visualization of the GN flakes of different sizes attached to the cell clump.

The morphology of the cells after incubation with GN, GO, and nGO was evaluated on living cultures without fixation using two different visualization systems and after fixation, and staining was done by May-Grünwald and Giemsa (MGG) method. In all cell lines, cells were attached to the GO flakes, which was especially visible in the living cultures, while cells were suspended (Figure 11, Figure 12, Figure 13, Figure 14, Figure 15 and Figure 16). After analyzing the live images in phase contrast, it also seems that the cells favorably attached to the thinnest flake rather than to the thickest, clearly visible ones (Figure 11 and Figure 12). The effect was not visible for larger GN flakes, only relatively smaller flakes were attaching, what was visible after MGG staining (Figure 13). The effect was also less visible in nGO than in the GO group. Moreover, characteristic shades were present in all GO-treated groups after MGG staining, confirming attachment of the cells to the GO flakes (Figure 11 and Figure 14). Very thick GO flakes were rarely visible in the cultures (Figure 15). In the DOHH-2 cell line, where cells grew in clumps, there were visible aggregates of all the graphene (Figure 16). Overall, attachments of the natural graphene were not as specific as the oxidized forms of graphene. Selected characteristic features are presented in the additional panels in Figure 11, Figure 12, Figure 13, Figure 14, Figure 15 and Figure 16.

## 4. Discussion

In the presented studies, we examined the effect of three nanomaterials belonging to the graphene family—namely GN, GO, and nGO—on six different cell lines derived from two divergent hematological malignancies. KMS-12-BM, H929, U266, and MM.1S cell lines are derived from MM patients with different genetic backgrounds, while KARPAS299 and DOHH-2 are derived from NHL lymphoma patients. The cell lines arise from either clonal plasma cells in the case of myelomas or from T/B lymphocytes in the case of lymphomas, resulting in their non-adherent characteristics during in vitro culturing. While the effect of carbon nanomaterials on cells cultured in monolayers or 3D tumor models has been broadly studied, the effects on non-adherent cells, derived from hematological cancers, are barely known. Based on the performance of basic metabolic tests, flow cytometric analysis of apoptosis induction and cell cycle, and broad morphological examination, we have demonstrated that GN, GO, and nGO have low to moderate cytotoxic effects, despite visible direct interactions between the nanostructures and the cells. The most interesting results were obtained for the oxidized forms of graphene, GO and nGO.

To evaluate the cytotoxic effect of GN, GO, and nGO against the cells, we performed two types of viability tests, obtaining different results. The first was the colorimetric MTT test, where yellow, soluble tetrazolium salt is converted into purple formazan crystals by mitochondrial dehydrogenases, predominantly succinate dehydrogenase, and the formation of crystals reflects the number of viable cells. The second was the luminometric ATP assay, where luciferin is oxidized by luciferase to oxyluciferin in the presence of ATP and light is generated. The luminescent signal is directly proportional to the amount of ATP released from viable cells during the lysis step. According to the results obtained from the MTT test, GN, GO, and nGO showed mild cytotoxic effects in the highest concentrations (50 and 100 mg/L) in the lymphoma cell lines and no toxic effect in myeloma cells, except for GO in MM.1S (Figure 6). MM.1S was the only cell line representing semi-adherent growth, meaning that the cells grew in a suspension, but part of them was lightly attached to the culture dish. The obtained results might be correlated with doubling time for particular cell lines since for lymphoma cell lines the time is 30 to 40 h (for KARPAS299 [33] and DOHH-2 [34], respectively), while for the myeloma cells it is from 55 to 80 h (55 h for U266 [35], 60 h for KSM-12-BM [36], 70–80 h for H929 [37], and 72 h for MM.1S [38]). Therefore, it could indicate a slight antiproliferative effect of graphene family materials. On the other hand, the results obtained from the ATP assay suggested a high toxic effect in all groups, starting from the lowest used concentration (Figure 7). Interestingly, the highest cytotoxic effect was observed for GO, while oxidized forms of graphene are generally more biocompatible, and GO had a low toxic effect against other cell lines, such as liver-derived cells, in our previous studies [23,39]. Furthermore, natural graphene or reduced forms of graphene oxides exhibited not only a cytotoxic effect against other cancer cells [17,40], but also more hemolytic character when compared to GO [41]. However, it was also found that GO and other carbon nanostructures, such as carbon nanotubes, might non-specifically inhibit enzymatic reactions by binding to the structure of a protein [24,39,42]. Considering the nature of the ATP assay, where the reaction should occur very rapidly [43], it may be possible that the obtained results are due to the mentioned interactions between large graphene oxide platelets and luciferase, which inhibited the oxidation of luciferin, or between the luciferin and GO. Another possibility is signal quenching by the presence of GO, which was highly hydrophilic and stable in a water medium (zeta potential close to −60 mV; Table 1); thus, it was not possible for it to be completely removed by simple centrifugation techniques. It was previously demonstrated by other authors that graphene and graphite oxides can quench fluorescence [44,45].

The results were not conclusive, and we further performed an analysis of apoptosis induction (Figure 8) and cell cycle evaluation (Figure 10) after treatment with a moderate dose of GN, GO, and nGO. The results confirmed no specific toxicity, which supports the results obtained from the MTT assays rather than from the ATP assays, indicating non-specific physicochemical interactions between graphene materials and ATP test components. Moreover, this confirms that carbon nanostructures have unique physicochemical properties, which results in difficulties and sometimes false results, while applying widely used standard cytotoxicity assays. For example, Jiao et al. indicate that the optical properties of graphene may be interference for many in vitro assays, including absorption and reflection effect, because graphene has prominent light absorption property [46]. Thereby, the results from the MTT assay could be overestimated, while for the ATP assay underestimated. To minimize the possibility of the assay’s compound interactions with the studied graphene family materials, we performed cell-free control experiments, obtaining similar results similar, verifying that MTT and WST-8 assays were reliable and free of interference [47]. Nevertheless, the possibility of the mentioned overestimation for MTT and underestimation for ATP cannot be completely excluded. Therefore, the apoptosis assay, cell cycle evaluation, and morphology evaluation helped to interpret the possible results. Interestingly, even though we did not observe significant apoptosis induction, we noticed great change in cellular granularity in nGO groups while analyzing diagrams for cytometric assays (Figure 9), which suggests nGO adhesion to the cell membrane or internalization of nGO within the cells.

Low toxicity was also confirmed visually by morphology evaluation, which was presented in this article through pictures taken during live imaging and after staining by the MGG method. We did not observe significant differences between control cells and cells incubated with graphene family materials, when considering the cell density and general morphology (Figure 11, Figure 12, Figure 13, Figure 14, Figure 15 and Figure 16). However, we observed differences between cell lines and treatment groups for cell distribution within the culture, which might explain differences in toxicity in non-adherent and adherent cell cultures. In the GN group, we observed that most of the thickest flakes settled on the bottom and only for the DOHH-2 cell line, which grows in small clumps. We also observed increased amounts of GN platelets in cells (Figure 16). In GO groups, cells were attached to the flakes while growing in the suspension. However, it was difficult to demonstrate in living cells that the attachment is still visible, especially on phase-contrast images (Figure 11 and Figure 12). Characteristic patterning around cell clumps was also observed after MGG staining. Interestingly, in DOHH-2 cells we observed a slight dispersion of the cells on GO flakes in comparison to the cell clumps observed in the control (Figure 16). In the nGO group, we observed both nanoplatelet settling on the bottom and attaching to cells. The observation corresponds to the general physicochemical properties of the examined materials. GN, being hydrophilic with low stability (Table 1), settled mostly on the bottom, creating a concentration gradient. Thus, it is probably more toxic to the cells that are adhered to the culture dish than cells in a suspension. GO is highly hydrophilic and can create a very stable dispersion, with a high surface area available for cells to interact, while nGO creates a less stable dispersion (Table 1) and the platelets are of far smaller size (Figure 1).

Interestingly, studies conducted by Russier et al. using graphene suggested that the toxic effect of graphene might be related to the immune cell type since the authors demonstrated that graphene caused necrosis only in myelomonocytic cells (arisen from monocytes) but not in other immune cell populations [18]. Since multiple myelomas and lymphomas arise from plasma cells and B/T/lymphocytes or natural killer (NK) cells, it is possible that they are not susceptible to graphene. Our results are also in accordance with previous findings by Wu et al., who demonstrated that GO alone had no toxic effect on multiple myeloma cells, but it significantly increased the toxic effect of doxorubicin [48]. Pelin et al. suggested that graphene and GOs are cytotoxic only in high concentrations (above 20 mg/mL) and after long exposure times, what is similar to our findings [49]. Low cytotoxicity and the interactive surface of graphene oxides create promising possibilities for use in drug delivery platforms. In some of the previous experiments, graphene oxide used as a scaffold stimulated the growth of some cells and inhibited other cells (cell cultures of different origin from chicken embryos) [50,51]. In other studies, it was demonstrated that graphene was up taken by cells [52,53]. Still, there is a remaining concern of systemic toxicity and biodistribution of graphene family materials. Distribution of nanostructures to bone marrow is barely studied so far; however, it has been proven that some nanocarriers can be accumulated in bones [54,55,56]. Recent studies by Tang et al. showed that TiO_2_ nanoparticles can be used as an effective, targeted platform for multiple myeloma imaging and treatment [57]. However, it has also been demonstrated that the biokinetics of nanoparticles significantly differ between widely studied rodent models and primates [58], considering bone marrow distributions. Therefore, further advanced studies are needed.

## 5. Conclusions

We studied the effect of graphene, graphene oxide, and nanographene oxide on multiple myeloma and lymphoma cell lines, revealing low to moderate cytotoxicity against the selected cell lines. Low toxicity, an active surface, and the possibility for cell binding make graphene oxide a promising component for drug delivery systems. We also revealed that the choice of metabolic assay has an important impact on the test outcome when investigating the cytotoxicity of graphene and graphene oxides. Further advanced studies are needed to explain the nature of interactions between graphene family materials and cells, consequently, to establish the potential applications for hematological malignancies.

## Figures and Tables

**Table 1:** Zeta potential and average hydrodynamic diameter of the graphene family materials used in the experiments. Results are presented as the mean ± standard deviation.

Parameter	GN	nGO	GO
Zeta potential (mV)	11.0 ± 0.62	−13.7 ± 0.46	−64.3 ± 2.31
Average hydrodynamic diameter (nm)	5210.7 ± 2001.17	1740.0 ± 186.20	1246.7 ± 39.40

## References

[1] Geim A.K., Novoselov K.S. (2007). The rise of graphene. Nat. Mater..

[2] Compton O.C., Nguyen S.T. (2010). Graphene Oxide, Highly Reduced Graphene Oxide, and Graphene: Versatile Building Blocks for Carbon-Based Materials. Small.

[3] Wang S., Ferrag C., Noroozifar M., Kerman K. (2020). Simultaneous Determination of Four DNA bases at Graphene Oxide/Multi-Walled Carbon Nanotube Nanocomposite-Modified Electrode. Micromachines.

[4] Czerniak-Reczulska M., Niedzielska A., Jędrzejczak A., Jȩdrzejczak A. (2015). Graphene as a Material for Solar Cells Applications. Adv. Mater. Sci..

[5] Fernández S., Mojena A.B., Pedrós J., Inés A., Fernández M., Arnedo I., González J.P., De La Cruz M., Sanz D., Vela A.M. (2019). Advanced Graphene-Based Transparent Conductive Electrodes for Photovoltaic Applications. Micromachines.

[6] Singh R., Hong S., Jang J. (2017). Label-free Detection of Influenza Viruses using a Reduced Graphene Oxide-based Electrochemical Immunosensor Integrated with a Microfluidic Platform. Sci. Rep..

[7] Seo G., Lee G., Kim M.J., Baek S.-H., Choi M., Ku K.B., Lee C.-S., Jun S., Park D., Kim H.G. (2020). Rapid Detection of COVID-19 Causative Virus (SARS-CoV-2) in Human Nasopharyngeal Swab Specimens Using Field-Effect Transistor-Based Biosensor. ACS Nano.

[8] Karahan H.E., Wiraja C., Xu C., Wei J., Wang Y., Wang L., Liu F., Chen Y. (2018). Graphene Materials in Antimicrobial Nanomedicine: Current Status and Future Perspectives. Adv. Healthc. Mater..

[9] Valentini F., Calcaterra A., Ruggiero V., Pichichero E., Martino A., Losi F., Bertuccini L., Antonaroli S., Mardente S., Zicari A. (2019). Functionalized Graphene Derivatives: Antibacterial Properties and Cytotoxicity. J. Nanomater..

[10] Ye S., Shao K., Li Z., Guo N., Zuo Y., Li Q., Lu Z., Chen L., He Q., Han H.-Y. (2015). Antiviral Activity of Graphene Oxide: How Sharp Edged Structure and Charge Matter. ACS Appl. Mater. Interfaces.

[11] Li J., Zhou C., Luo C., Qian B., Liu S., Zeng Y., Hou J., Deng B., Sun Y., Yang J. (2019). N-acetyl cysteine-loaded graphene oxide-collagen hybrid membrane for scarless wound healing. Theranostics.

[12] Jian Z., Wang H., Liu M., Chen S., Wang Z., Qian W., Luo G., Xia H. (2020). Polyurethane-modified graphene oxide composite bilayer wound dressing with long-lasting antibacterial effect. Mater. Sci. Eng. C.

[13] Fusco L., Gazzi A., Peng G., Shin Y., Vranic S., Bedognetti D., Vitale F., Yilmazer A., Feng X., Fadeel B. (2020). Graphene and other 2D materials: A multidisciplinary analysis to uncover the hidden potential as cancer theranostics. Theranostics.

[14] Krasteva N., Keremidarska-Markova M., Hristova-Panusheva K., Andreeva T.D., Speranza G., Wang D., Draganova-Filipova M., Miloshev G., Georgieva M. (2019). Aminated Graphene Oxide as a Potential New Therapy for Colorectal Cancer. Oxidative Med. Cell. Longev..

[15] Rahman M., Ahmad M.Z., Ahmad J., Firdous J., Ahmad F.J., Mushtaq G., Kamal M.A., Akhter S. (2015). Role of Graphene Nano-Composites in Cancer Therapy: Theranostic Applications, Metabolic Fate and Toxicity Issues. Curr. Drug Metab..

[16] Ou L., Song B., Liang H., Liu J., Feng X., Deng B., Sun T., Shao L. (2016). Toxicity of graphene-family nanoparticles: A general review of the origins and mechanisms. Part. Fibre Toxicol..

[17] Jaworski S., Strojny B., Sawosz E., Wierzbicki M., Grodzik M., Kutwin M., Daniluk K., Chwalibog A. (2019). Degradation of Mitochondria and Oxidative Stress as the Main Mechanism of Toxicity of Pristine Graphene on U87 Glioblastoma Cells and Tumors and HS-5 Cells. Int. J. Mol. Sci..

[18] Russier J., León V., Orecchioni M., Hirata E., Virdis P., Fozza C., Sgarrella F., Cuniberti G., Prato M., Vázquez E. (2017). Few-Layer Graphene Kills Selectively Tumor Cells from Myelomonocytic Leukemia Patients. Angew. Chem. Int. Ed..

[19] Akhavan O., Ghaderi E., Hashemi E., Rahighi R. (2014). Ultra-sensitive detection of leukemia by graphene. Nanoscale.

[20] Strojny B., Kurantowicz N., Sawosz E., Grodzik M., Jaworski S., Kutwin M., Wierzbicki M., Hotowy A., Lipinska L., Chwalibog A. (2015). Long Term Influence of Carbon Nanoparticles on Health and Liver Status in Rats. PLoS ONE.

[21] Gurunathan S., Kang M.H., Jeyaraj M., Kim J.-H. (2019). Differential Cytotoxicity of Different Sizes of Graphene Oxide Nanoparticles in Leydig (TM3) and Sertoli (TM4) Cells. Nanomaterials.

[22] Gies V., Lopinski G., Augustine J., Cheung T., Kodra O., Zou S. (2019). The impact of processing on the cytotoxicity of graphene oxide. Nanoscale Adv..

[23] Strojny B., Sawosz E., Grodzik M., Jaworski S., Szczepaniak J., Sosnowska M., Wierzbicki M., Kutwin M., Orlińska S., Chwalibog A. (2018). Nanostructures of diamond, graphene oxide and graphite inhibit CYP1A2, CYP2D6 and CYP3A4 enzymes and downregulate their genes in liver cells. Int. J. Nanomed..

[24] El-Sayed R., Bhattacharya K., Gu Z., Yang Z., Weber J.K., Li H., Leifer K., Zhao Y., Toprak M.S., Zhou R. (2016). Single-Walled Carbon Nanotubes Inhibit the Cytochrome P450 Enzyme, CYP3A4. Sci. Rep..

[25] De Melo-Diogo D., Lima-Sousa R., Alves C.G., Correia I.J., Correia L. (2019). Graphene family nanomaterials for application in cancer combination photothermal therapy. Biomater. Sci..

[26] Kyle R.A., Rajkumar S.V. (2009). Treatment of Multiple Myeloma: A Comprehensive Review. Clin. Lymphoma Myeloma.

[27] Avet-Loiseau H. (2019). Introduction to a review series on advances in multiple myeloma. Blood.

[28] Pawlyn C., Davies F.E. (2019). Toward personalized treatment in multiple myeloma based on molecular characteristics. Blood.

[29] Jiang M., Bennani N.N., Feldman A.L. (2017). Lymphoma classification update: T-cell lymphomas, Hodgkin lymphomas, and histiocytic/dendritic cell neoplasms. Expert Rev. Hematol..

[30] Majchrzycki Ł., Augustyniak-Jabłokow M.A., Strzelczyk R., Maćkowiak M. (2015). Magnetic Centres in Functionalized Graphene. Acta Phys. Pol. A.

[31] Kurantowicz N., Strojny B., Sawosz E., Jaworski S., Kutwin M., Grodzik M., Wierzbicki M., Lipińska L., Mitura K., Chwalibog A. (2015). Biodistribution of a High Dose of Diamond, Graphite, and Graphene Oxide Nanoparticles After Multiple Intraperitoneal Injections in Rats. Nanoscale Res. Lett..

[32] Wierzbicki M., Jaworski S., Kutwin M., Grodzik M., Strojny B., Kurantowicz N., Zdunek K., Chodun R., Chwalibog A., Sawosz E. (2017). Diamond, graphite, and graphene oxide nanoparticles decrease migration and invasiveness in glioblastoma cell lines by impairing extracellular adhesion. Int. J. Nanomed..

[33] ECACC General Cell Collection: KARPAS 299. https://www.phe-culturecollections.org.uk/products/celllines/generalcell/detail.jsp?refId=06072604&collection=ecacc_gc.

[34] DOHH-2. https://www.dsmz.de/collection/catalogue/details/culture/ACC-47.

[35] U-266. https://www.dsmz.de/collection/catalogue/details/culture/ACC-9.

[36] KMS-12-BM. https://www.dsmz.de/collection/catalogue/details/culture/ACC-551.

[37] NCI-H929. https://www.dsmz.de/collection/catalogue/details/culture/ACC-163.

[38] MM.1S (ATCC® CRL-2974TM). https://www.lgcstandards-atcc.org/Products/All/CRL-2974.aspx.

[39] Sekretarska J., Szczepaniak J., Sosnowska M., Grodzik M., Kutwin M., Wierzbicki M., Jaworski S., Bałaban J., Daniluk K., Sawosz E. (2019). Influence of Selected Carbon Nanostructures on the CYP2C9 Enzyme of the P450 Cytochrome. Materials.

[40] Szczepaniak J., Strojny B., Sawosz E., Jaworski S., Jagiello J., Winkowska-Struzik M., Szmidt M., Wierzbicki M., Sosnowska M., Bałaban J. (2018). Effects of Reduced Graphene Oxides on Apoptosis and Cell Cycle of Glioblastoma Multiforme. Int. J. Mol. Sci..

[41] Jaworski S., Hinzmann M., Sawosz E., Grodzik M., Kutwin M., Wierzbicki M., Strojny B., Vadalasetty K.P., Lipińska L., Chwalibog A. (2017). Interaction of different forms of graphene with chicken embryo red blood cells. Environ. Sci. Pollut. Res..

[42] Sun X., Feng Z., Hou T., Li Y. (2014). Mechanism of Graphene Oxide as an Enzyme Inhibitor from Molecular Dynamics Simulations. ACS Appl. Mater. Interfaces.

[43] CellTiter-Glo® Luminescent Cell Viability Assay. https://pl.promega.com/-/media/files/resources/protocols/technical-bulletins/0/celltiter-glo-luminescent-cell-viability-assay-protocol.pdf?la=en.

[44] Liu Y., Liu C., Liu Y. (2011). Investigation on fluorescence quenching of dyes by graphite oxide and graphene. Appl. Surf. Sci..

[45] Povedailo V.A., Ronishenko B.V., Stepuro V.I., Tsybulsky D.A., Shmanai V.V., Yakovlev D.L. (2018). Fluorescence Quenching of Dyes by Graphene Oxide. J. Appl. Spectrosc..

[46] Jiao G., Qiu J., Xu H., He X., Li X., Zhang N., Liu S. (2015). Limitations of MTT and CCK-8 assay for evaluation of graphene cytotoxicity. RSC Adv..

[47] Chng E.L.K., Chua C.K., Pumera M. (2014). Graphene oxide nanoribbons exhibit significantly greater toxicity than graphene oxide nanoplatelets. Nanoscale.

[48] Zhao X., Zhao C., Wang Y., Li Y., Du L., Cui Z., Wu S. (2014). Cytotoxicity of graphene oxide and graphene oxide loaded with doxorubicin on human multiple myeloma cells. Int. J. Nanomed..

[49] Pelin M., Fusco L., León V., Martín C., Criado A., Sosa S., Vázquez E., Tubaro A., Prato M. (2017). Differential cytotoxic effects of graphene and graphene oxide on skin keratinocytes. Sci. Rep..

[50] Wierzbicki M., Hotowy A., Kutwin M., Jaworski S., Bałaban J., Sosnowska M., Wójcik B., Wędzińska A., Chwalibog A., Sawosz E. (2020). Graphene Oxide Scaffold Stimulates Differentiation and Proangiogenic Activities of Myogenic Progenitor Cells. Int. J. Mol. Sci..

[51] Zielińska-Górska M., Hotowy A., Wierzbicki M., Bałaban J., Sosnowska M., Jaworski S., Strojny B., Chwalibog A., Sawosz E. (2020). Graphene oxide nanofilm and the addition of l-glutamine can promote development of embryonic muscle cells. J. Nanobiotechnol..

[52] Sawosz E., Jaworski S., Kutwin M., Hotowy A., Wierzbicki M., Grodzik M., Kurantowicz N., Strojny B., Lipińska L., Chwalibog A. (2014). Toxicity of pristine graphene in experiments in a chicken embryo model. Int. J. Nanomed..

[53] Jaworski S., Sawosz E., Kutwin M., Wierzbicki M., Hinzmann M., Grodzik M., Winnicka A., Lipińska L., Wlodyga K., Chwalibog A. (2015). In vitro and in vivo effects of graphene oxide and reduced graphene oxide on glioblastoma. Int. J. Nanomed..

[54] Sago C.D., Lokugamage M.P., Islam F., Krupczak B.R., Sato M., Dahlman J.E. (2018). Nanoparticles That Deliver RNA to Bone Marrow Identified by in Vivo Directed Evolution. J. Am. Chem. Soc..

[55] Zong H., Sen S., Zhang G., Mu C., Albayati Z.F., Gorenstein D.G., Liu X., Ferrari M., Crooks P.A., Roboz G.J. (2015). In vivo targeting of leukemia stem cells by directing parthenolide-loaded nanoparticles to the bone marrow niche. Leukemia.

[56] Sou K. (2012). Advanced drug carriers targeting bone marrow. Recent Advances in Novel Drug Carrier Systems.

[57] Tang R., Zheleznyak A., Mixdorf M., Ghai A., Prior J., Black K.C.L., Shokeen M., Reed N., Biswas P., Achilefu S. (2020). Osteotropic Radiolabeled Nanophotosensitizer for Imaging and Treating Multiple Myeloma. ACS Nano.

[58] Chiarelli P.A., Revia R.A., Stephen Z.R., Wang K., Jeon M., Nelson V., Kievit F.M., Sham J., Ellenbogen R.G., Kiem H.-P. (2017). Nanoparticle Biokinetics in Mice and Nonhuman Primates. ACS Nano.

